# Medical Society of the County of Albany

**Published:** 1879-02

**Authors:** T. K. Perry

**Affiliations:** Secretary


*ART. III.—Medical Society of the County of Albany. Semi-Monthly Meeting Nov. 20th,* 1878. Reported by T. K. Perry, M. D., Secretary.

The Society met at the usual hour and place at the City Hall, in Albany.

The President, Dr. F. C. Curtis called the meeting to order, and the minutes of the previous meeting having been read by the Secretary, and duly approved, the report of the committee on the propositions and suggestions contained in the Presidents "Inaugural Address" was then submitted to the Society by their chairman, Dr. A. Van Der Veer.

The report is substantially as follows: In relation to the committee on Hygiene, that the present committee if still in existence together with such additions as may be necessary, be instructed to carry out as far as possible the suggestions of the president. Also that the committee shall be composed of not less than five or more than seven members. The committee on Pathology, or Clinic Pathology, with sub-committee on Microscopy, would prove one of the most useful bodies ever called into existence by this society. Its members should however consist of earnest, active men, who are willing to work, and would prepare the material given to them for its presentation to the society. A committee of nine members would perhaps suffice, and sub-committee might be formed for the care of special subjects or departments. In relation to "Nominating Committee;" that the number shall consist of nine instead of five, and that seven shall constitute a quorum.

It reads as follows:

*Resolved,* That at the semi-annual meeting, a committee be elected for the purpose of nominating a list of the officers to be voted for at the ensuing annual meeting, which shall be known as the regular ticket, and that the vote for this committee be by ballot, each member voting for but one person, and the gentlemen receiving the nine highest votes shall be the committee.

*Further,* That the committee shall give notice to each member of the society, through the Secretary, of the ticket upon which they shall agree, two weeks before the annual meeting.

*Resolved,* That the annual dues be reduced from $2,00 to $1,00, and that the delegates to State Society, from this society shall be requested to pay the annual dues of $20, due the State Society.

Your committee would respectfully suggest that the attention of the board of Censors be directed to the law now in force respecting the preliminary examination of medical students previous to entering the offices of practitioners.

In regard to registration; that we follow the suggestions of the State Society, therefore be it

*Resolved,* That a committee of three, to be known as the *Committee on registration* be appointed, one of whom shall be the Secretary, whose duty it shall be to perform the duties specified by the State Society, and also to have in charge the register and chronological list of the society for the pnrpose of making such corrections and additions to it as may be necessary from time to time under the various heads, and further to publish the annual register of the society, the same to be done by these without expense to the society.

A. VAN DER VEER,<br>

HENRY MARCH,<br>

WM. HAILES, Jr., } Committee.

A general discussion ensued relative to the propriety of adopt-the report in toto, but the opinions were so divided that the president ruled, that as the resolution offered regarding a reduction of the annual dues contemplated an amendment of the By-Law, it would be regarded simply as a notice of amendment, and would lie over till the annual meeting.

The remainder of the report was accepted and adopted.

Dr. S. H. Sabine then presented a specimen of what was believed to be true rupture of the uterus, the breach of continuity extending across the entire fundus. The organ was about the size of one at the third month of pregnancy, and appeared in most respects healthy. The tissue at the point of separation while showing very little evidence of inflammatory action, or deposit of a malignant character, was nevertheless very much thined and edges ragged.

The history of the case of which I give a synopsis, was as follows:

Mrs. S——, æt. 46, mother of eight children, the youngest nearly four years old, presented herself at the Dr's office, August 26th, 1878, and requested treatment for an excessively irritable stomach accompanied with nausea and vomiting. She could not believe she was pregnant, yet had not menstruated for three months. Had always been quite regular, and had never knowingly suffered from any uterine complaint.

Believing the case to be one of gastric irritation solely, she was treated accordingly, but failing to obtain the desired relief she sought advice elsewhere, and the Doctor saw no more of her until October 4th, of same year, at which time he was called to see her at her home, she being confined to her bed and flowing freely. The usual treatment was resorted to with the effect of very materially lessening the discharge.

The clots which came away were examined closely, but nothing in any way resembling a fœtus was to be found.

October 5th. Flowing quite freely again and much prostrated. Tampan applied and ergot and brandy internally—catheter employed to empty bladder—flowing checked.

Oct. 6th Was called about 5 A. A., to see patient who was in great pain across the lower part of abdomen. Morphia hypodermically, and tampon removed. 11 A. M.—patient sinkingly rapidly—stimulants were given freely but she did not rally, and at 3 P. M. expired.

Post Mortem twenty-five hours after death—body well nourished, skin very pale, cuticle gives way on slightest pressure, abdomen distended with gas, uterus about the size of one at three months of pregnancy—empty and ruptured across entire fundus, the opening communicating with the general peritoneal cavity, remaining organs healthy—no blood in abdominal cavity, very little pus and no evidence of peritonitis.

Dr. Burton of Troy, who had seen and treated this patient subsequent to her first visit at Dr. Sabine's, states that while she was under his care, his treatment being for gastric derangement merely, he was called in haste one day to see her, and to his surprise found her in great pain across the abdomen and flowing freely. An examination revealed, projecting from the vulva, a polypus about the size of a fig, and evidently having its attachment within the uterus. It came away during the examination. The hemorrhage was subdued, uterus contracted nicely, and she made an apparent good recovery. During the interval between the expulsion of this body and her death, she had been gaining in health and strength, and on the evening preceeding her death had been in unusual good spirits.

In conclusion, Dr. Sabine expressed his inability to account satisfactorily for all the conditions which the case presented. Did not believe it had been one of the varieties of pregnancy, although Prof. T. Gaillard Thomas, of New York, who had been written to concerning the affair, believed at first it had been one of Extra Uterine Pregnancy, the fœtal body escaping into the abdomen and missing observation at the post mortem. The substance removed by Dr. Burton he thought was probably a portion of the Membrance Decidur.

In a subsequent letter however, he admitted too much haste in diagnosis, and thought after all, it might not have been any form of pregnancy.

On the completion of the paper, Dr. Curtis very briefly summied up the more important points and requested a thorough discussion as the case was both unique and interesting.

### DISCUSSION.

Doctors Beckett, Blatner, Cook and Hailes participated in the discussion, they all believed that a body of some nature had evidently been expelled from the womb, as in no other way did they believe the hemorrhage and contractile pains could be accounted for. As to the exact nature of this body there was a division of opinion, some believing it to have been of fœtal origin, the remainder thought more probably, polypoid. An important question which several members addressed to Dr. Sabine was: What was the quantity, and in what way did he explain the presence of the pus which he stated was found in the abdomen after death? As to the quantity the reply was, a very little, less than a teaspoonful probably—concerning the cause he plead entire ignorance, as well also inability to even conjecture.

The President at this time ordered a recess for refreshments. After reassembling, Dr. J. H. Blatner presented a specimen which he believed to be a true *diphtheritic cast of the trachea*, and the following is the history of the case:

Patient, a girl æt. eight years. When first seen had been complaining for several days. Deposit on fauces well marked and constitutional symptoms somewhat alarming.

Diagnosed diphtheria and treated both locally and constitutionally.

The disease however had obtained so firm a hold that notwithstanding recourse was had to any measure by which relief might be obtained, it steadily increased in severity, ultimately proving fatal. The period between apparent inception and death in this case was nine days.

The post mortem revealed besides the specimen exhibited a deposit of a somewhat similar nature extending down the bronchii even (the Dr. believed) to those of the smaller order.

DISCUSSION.

Dr. Curtis in referring to the case, laid especial stress upon the well recognised tendency of the diphtheritic process upward rather than downward, the latter being more especially peculiar to croupous exudations. The Dr. he thought, had not made sufficient mention of this in his recital. Believed the presence of albumen in the urine a diagnostic point to distinguish between diphtheria and croup. Inquired of Dr. J. P. Boyd, Jr., whether in his experience this were not the fact—Dr. Boyd thought not in all cases.

Dr. Boyd Jr. remembered a case in which Dr. E. S. Hun wished him to see a child four years of age which he was attending at the St. Vincents Asylum. It was supposed the child had swallowed some foreign body, as in no other way could the nurse account for its labored breathing. Believed at the time it was a case of diphtheria.

The child died, and on examination there was found, extending from the glottis to bifurcation of bronchii a deposit in every way resembling that of diphtheria.

The peculiar feature in this case was the entire absence of deposit above the glottis.

Dr. Thomas Beckett said he had every reason to believe that an epidemic of true diphtheria was prevalent in the western part of the city, as he had been called to see a number of patients living within an area of half a square mile or more, all of whom seemed to be somewhat similarly affected. In each case the deposit on fauces was well shown and constitutional symptoms evident. His treatment was both local and constitutional—Sal. Acid Salicylic to throat. Terrum, Quinia and Potassa Chloras internally.

His per centage of losses was very small. Believes the throat affection merely a local manifestation of a constitutional disorder.

Dr. A. T. Van Vranken related a case of diphtheria complicated with Subleola. The entire eruption which was quite profuse, disappeared in twenty-four hours time, and the prostration, which was before very great, seemed now to be increased, and after a few hours the trachea filled and patient expired.

Doctors Boyd, Blatner, Cook and Perry, engaged in a discussion relative to the peculiarity of the diphtheritic exudate and the appearance of the underlying surfaces following its removal.

Dr. Cook, quoting from Ziemssen, said he believed the germ theory to be the accepted one, and in regard to the membrane, thought Oertel distinctly stated that its separation occasioned neither a ragged, bloody or ulcerated surface underneath.

The others thought its removal, (unless by absorption) meant a loss of substance and a consequently more or less ulcerated surface remaining.

Following this discussion Dr. A. Van Der Veer presented for the inspection of the society, a specimen of more than ordinary interest. It was that of a testicle very much enlarged and bearing evidences of a malignant deposit. He had removed it from a young man of twenty-five, who was in all other respects healthy and sound.

The patient stated that while frolicking he had received an injury at this point, and the pain and swelling had increased rapidly. There was an interval of thirteen weeks between the accident and operation. The Dr. believed it to be Encephaloid, but pending microscopical examination withheld a positive diagnosis.

There being no further business before the Society it was declared adjourned.

